# Self-Harm Among 17-Year-Old Adolescents With/Without Disabilities in the United Kingdom

**DOI:** 10.1027/0227-5910/a000951

**Published:** 2024-03-15

**Authors:** Eric Emerson, Zoe Aitken, Joanne Arciuli, Tania King, Gwynnyth Llewellyn, Anne Kavanagh

**Affiliations:** ^1^ Centre for Disability Research, Faculty of Health and Medicine, Lancaster University, UK; ^2^ Centre for Disability Research and Policy, Faculty of Medicine and Health, University of Sydney, NSW, Australia; ^3^ College of Nursing and Health Sciences, Flinders University, Bedford Park, SA, Australia; ^4^ Melbourne School of Population and Global Health, University of Melbourne, VIC, Australia

**Keywords:** disability, self-harm, suicide attempts, inequalities

## Abstract

**Abstract:** *Background:* Self-harm is a critical public health issue for adolescents/young adults. *Aims:* To estimate the prevalence of self-harm among adolescents with/without disabilities in the United Kingdom. *Method:* Secondary analysis of data collected at age 17 in the UK’s Millennium Cohort Study. *Results:* Prevalence of self-harm was significantly greater among adolescents with disabilities for suicide attempts and six forms of self-harming behaviors. The lifetime prevalence of suicide attempts was 5.3% (4.5–6.3) among adolescents without disabilities, 21.9% (18.2–26.2) among adolescents with less limiting disabilities, and 25.5% (17.2–35.9) among adolescents with more limiting disabilities. Adjusted prevalence rate ratios ranged from 5.13 (3.58–7.36) for those with mental health limitations to 1.48 (0.65–3.35) for those with mobility limitations. Similar patterns were observed for the 12-month prevalence of six self-harming behaviors. *Limitations:* Further studies are needed to identify potential mediators of the association between disability and self-harm that are potentially modifiable. *Conclusion:* Adolescents with disabilities are at markedly greater probability of suicide attempts and self-harming behaviors than their peers.

Self-harm (an umbrella term that we use to encompass suicide, suicide attempts, and nonsuicidal self-injury) is an increasing public health problem (Borschmann & Kinner, 2019). A recent meta-analysis estimated that the global 12-month prevalence of self-harm in children and adolescents was 14.2% (Lim et al., 2019), and evidence from the United Kingdom suggests that children and adolescents who presented to hospital following an episode of self-harm were 30 times more likely to die by suicide within a year compared to the general population (Hawton et al., 2020). As such, preventing self-harm is a critical issue in adolescent health. Interventions need to address the situation of groups who are at particular risk of self-harm. Girls and women and people with disabilities are increasingly seen as two of such groups (Krahn et al., 2015). Population-based studies have reported higher rates of self-harm among adults with disabilities when compared with their peers (Khazem, 2018; Khurana et al., 2021; Marlow et al., 2022; Milner et al., 2019). However, little is known about the prevalence of, and risk factors associated with, self-harm among adolescents with disabilities (Emerson et al., 2019). Three population-based studies have reported conflicting results, with the prevalence of self-harm being significantly greater among adolescents with disabilities when compared with their peers in the United Kingdom and Oregon, USA (Emerson et al., 2019; Tejera et al., 2019) but no such differences being found in Australia (King et al., 2019). The aim of the present paper was to provide additional population data on the prevalence and risk factors for self-harm for adolescents with and without disabilities. Our purpose was to further explore the factors associated with increased self-harm within a group of adolescents with disabilities which, in turn, could direct targeted public health interventions for this group.

## Method

We conducted secondary analysis of data collected from adolescents at ages 14 and 17 years the most recent of the UK’s birth cohort studies, the *Millennium Cohort Study* (https://cls.ucl.ac.uk/cls-studies/millennium-cohort-study/; Fitzsimons et al., 2020). Child Benefit Records, a non-mean tested benefit with a near universal coverage of UK children, were used to randomly select participants. At age 14, information was collected from parental informants on 11,726 adolescents and from 10,971 adolescent participants. At age 17, information was collected from 9,528 adolescent informants. Data used in the present analyses were collected by computer-assisted personal interview with a parental informant and, separately, computer assisted self-interview or personal interview with the adolescent.

Disability at age 17 was defined as (1) giving an affirmative answer to the question “Do you have any physical or mental health conditions or illnesses lasting or expected to last 12 months or more?” and (2) responding that “These condition(s) or illness(es) reduce [their] ability to carry out day-to-day activities?” *a lot* or *a little*. We used responses to the second question to distinguish between more limiting disability (reduced ability *a lot*) and less limiting disability (reduced ability *a little*). Information was also collected on the presence of nine categories of functional impairment type (*vision, hearing, mobility, dexterity, learning, memory, mental health, stamina, social/behavioral, other;* see Supplementary Materials for more detail). Disability data were missing for 0.1% of respondents.

The lifetime prevalence of suicide attempts (up to age 17) was assessed using a binary question (“Have you ever hurt yourself on purpose in an attempt to end your life?”). In addition, binary questions were asked of all participants related to whether the respondent had engaged in six specific self-harming behaviors in the last year (*taken an overdose of tablets, cut or stabbed self, burned self, bruised or pinched self, pulled out your hair, hurt yourself some other way).* While some of these behaviors (e.g., *bruised or pinched self, pulled out your hair*) are almost certainly to have been examples of nonsuicidal self-injury, others (e.g., *taken an overdose of tablets*) could have been suicide attempts. However, given that suicidal ideation was not measured, we will refer to all six of these as self-harming behaviors. Self-harm data were missing for 2.5% of respondents.

Covariates included potential confounders (sex [male/female], ethnicity [White/non-White], family income poverty [yes/no]) and in subsequent analyses potential risk factors for self-harm (sexual orientation [heterosexual/other], exposure to bullying [yes/no], and scoring above the cut-off on a self-report measure of depression at age 14). Details of the derivation of covariates and potential risk factors are presented in Electronic Supplementary Material 1 (ESM 1).

Analyses were undertaken in Stata 16 using inverse probability weights to address sampling and attrition biases and survey data analysis options to address clustering in survey design. Adjusted prevalence rate ratios were estimated controlling for the potential confounding effects of sex, ethnicity, and exposure to family income poverty.

## Results

12.4% (95% CI 10.8%–14.1%) of respondents were identified as having a disability, 7.7% (95% CI 6.7%–8.7%) with a less limiting disability, and 4.7% (95% CI 3.6%–6.1%) with a more limiting disability. Characteristics of the sample are presented in Table E1 in ESM 1. Prevalence of self-harm among participants with/without disability with adjusted prevalence rate ratios to estimate the probability of self-harm among participants with disability are presented in Table 1. Prevalence of self-harming behaviors in the previous year was markedly greater among adolescents with disabilities for all six self-harming behaviors compared to those without disabilities. The lifetime prevalence of attempted suicide was 3.67 times and 3.87 times greater for adolescents with less and more limiting disability, respectively, compared to those without disability. Lifetime prevalence of suicide attempts and 12-month prevalence of self-harming behaviors was greater among adolescents with more limiting disabilities than among adolescents with less limiting disabilities (though the 95% confidence intervals between those with more and less limiting disabilities were overlapping).

**Table 1:** Lifetime prevalence of suicide attempts and 12-month prevalence of engaging in specific self-harming behaviors among adolescents with more limiting disability, less limiting disability, and no disability with adjusted prevalence rate ratios to estimate the probability of self-harm among participants with more limiting and less limiting disability

Behavior	Prevalence (with 95% CI)			Adjusted prevalence rate ratio (APRR)	
	With more limiting disability	With less limiting disability	Without disabilities	More limiting disability	Less limiting disability
Ever hurt themselves on purpose in an attempt to end life	25.5% (17.2–35.9)	21.9% (18.2–26.2)	5.3% (4.5–6.3)	3.87*** (2.31–6.51)	3.67*** (2.94–4.57)
Participation in specific self-harming behaviors (including nonsuicidal self-harm) in the last year					
Taken an overdose of tablets	14.6% (9.1–22.4)	10.8% (8.5–13.8)	1.7% (1.3–2.3)	7.17*** (3.97–12.98)	5.73*** (4.00–8.22)
Cut or stabbed self	30.2% (21.4–40.7)	26.5% (22.2–31.4)	9.1% (7.9–10.4)	2.87*** (1.84–4.48)	2.70*** (2.18–3.35)
Burned self	18.7% (12.0–27.8)	11.1% (8.4–14.6)	4.1% (3.1–5.5)	3.85*** (2.02–7.33)	2.48*** (1.58–3.88)
Bruised or pinched self	35.2% (24.4–47.8)	27.1% (22.6–32.2)	12.9% (11.5–14.5)	2.55*** (1.75–3.72)	2.02*** (1.65–2.47)
Pulled out hair	23.6% (16.1–33.2)	15.7% (12.6–19.6)	5.8% (4.8–6.9)	3.57*** (2.18–5.83)	2.52*** (1.91–3.34)
Hurt themselves some other way	17.5% (11.3–26.2)	9.7% (7.4–12.6)	3.8% (3.0–4.9)	4.65*** (2.89–7.49)	2.54*** (1.78–3.62)
One or more self-harming behavior in the last year	42.8% (30.2–56.3)	41.1% (35.3–47.1)	21.1% (19.4–23.0)	1.88** (1.31–2.67)	1.86*** (1.59–1.17)
*Note*. APRR adjusted for sex, ethnicity, and exposure to family income poverty.					
***p < .001.					

Variation by type of functional impairment was investigated for suicide attempts (lifetime) and each form of self-harming behavior undertaken in the previous year. Adjusted prevalence rate ratios (adjusted for sex, ethnicity, and family income poverty) for attempted suicide were 5.13 (3.58–7.36) for mental health, 3.64 (2.72–4.88) for learning/understanding, 3.62 (1.51–8.68) for hearing, 3.34 (2.40–4.64) for memory, 2.98 (1.50–5.91) for social/behavioral, 2.75 (1.89–4.01) for stamina, 2.14 (0.58–7.82) for vision, 1.75 (1.01–3.03) for dexterity, 1.48 (0.65–3.35) for mobility. Adjusted prevalence rate ratios for specific forms of self-harming behaviors are presented in Table E2 in ESM 1. The prevalence of specific forms of self-harming behaviors in the previous year was particularly high for adolescents with functional impairments associated with mental health and learning/understanding.

Potential risk factors for suicide attempts (lifetime) and self-harming behaviors (previous year) among adolescents with disability are presented in Table E3 in ESM 1. Lifetime probability of suicide attempts was significantly greater for females, adolescents with nonheterosexual sexual orientation, those exposed to family poverty, and those scoring above the cut-off on a self-report measure of depression at age 14. Significantly increased probability of self-harming behaviors in the previous year was predicted by scoring above the cut-off on a self-report measure of depression at age 14 and nonheterosexual sexual orientation (all six forms of self-harming behaviors); female sex (5 forms); more limiting disability, having been bullied and majority ethnic status (2 forms); and family poverty (1 form). Similar associations (as evidenced by the marked overlap of confidence intervals between groups) were observed among adolescents without disability (Table E4 in ESM 1).

## Discussion

The results indicate that adolescents with disabilities, especially those with functional impairments associated with mental health and learning/understanding, have a particularly high probability of attempted suicide (lifetime) and engaging in a range of specific self-harming behaviors (previous year). There was also a consistent pattern of higher prevalence of self-harm among adolescents with more limiting disabilities when compared with those with less limiting disabilities.

Notable risk factors for self-harm among adolescents with disabilities (e.g., female sex, other sexual orientation, being bullied, depression at age 14) were similar to those without disabilities and suggest opportunities for targeting (girls) and preventative interventions (bullying reduction, mental health promotion). However, further studies are needed to identify potential mediators of the association between disability and self-harm that are potentially modifiable. These include both risk of depression and exposure to bullying, both of which are important risk factors for suicide and have been found to be more common among people with disabilities in other studies (Emerson et al., 2019; Kavanagh et al., 2018; King et al., 2018; King et al., 2019).

Public heath approaches to reducing self-harm among adolescents will need to make reasonable accommodations to policies and practices to ensure that they are equally accessible and effective for adolescents with disabilities. Codesigning public health approaches with people who have lived experience of disability must be a priority.

## Electronic Supplementary Material

The electronic supplementary material is available with the online version of the article at https://doi.org/10.1027/0227-5910/a000951

**ESM 1.** Disability questions and Tables E1–E3.

## Notes

**Publication Ethics:** This study was performed in line with the principles of the Declaration of Helsinki. Ethical approval for data collection and dissemination was obtained from the National Research Ethics Service Research Ethics Committee North East (ref: 17/NE/0341). Informed consent was obtained from all individual participants included in the study.

**Open Data:** The data underlying this article are available through the UK Data Service at https://ukdataservice.ac.uk/.
