# Supplementary material for: Self-Harm Among 17-Year-Old Adolescents With/Without Disabilities in the United Kingdom
Source: Crisis. 2024 Mar 15;45(5):376–9. doi: 10.1027/0227-5910/a000951 (PMC11423416; doi:10.1027/0227-5910/a000951)
Supplement: Supplementary file 1 [file cri_45_5_376_esm1.pdf]

https://doi.org/10.1027/0227-5910/a000951
© 2024 The Author(s). Distributed as a Hogrefe OpenMind article under the license CC BY 4.0 (https://creativecommons.org/licenses/by/4.0)

Electronic Supplementary Material 1 (ESM 1)

Disability Questions

1. *Do you have any physical or mental health conditions or illnesses lasting or expected to last 12 months or more?*
2. *Does this/Do any of these condition(s) or illness(es) affect you in any of the following areas?*
    a. *Vision (for example blindness or partial sight)*
    b. *Hearing (for example deafness or partial hearing)*
    c. *Mobility (for example walking short distances or climbing stairs)*
    d. *Dexterity (for example lifting and carrying objects, using a keyboard)*
    e. *Learning or understanding or concentrating*
    f. *Memory*
    g. *Mental health*
    h. *Stamina or breathing or fatigue*
    i. *Socially or behaviourally (for example associated with autism, attention deficit disorder or Asperger's Syndrome)*
    j. *Other*
3. *Does this/Do any of these condition(s) or illness(es) reduce your ability to carry out day-to-day activities? Would you say…* (a lot, a little, not at all)

Note: Respondents were allowed to select as many categories as necessary in response to Q2.

Sex

Information on sex was collected at early Waves in MCS. This was recoded into a binary variable (male/female) No data were missing for Wave 7 respondents for whom valid disability and self-harm data were available.

Ethnicity

Detailed information on ethnicity was collected at multiple Waves in MCS. Given the very small numbers in some cells, this was recoded into a binary variable (ethic minority), with 'white' coded as 0 and all other response options coded as 1. No data were missing for Wave 7 respondents for whom valid disability and self-harm data were available.

Sexual Orientation

Sexual orientation was primarily derived from responses to the following question.

- *Which of the following options best describes how you currently think of yourself?*
    - *Completely heterosexual / straight*
    - *Mainly heterosexual / straight*
    - *Bisexual*
    - *Mainly gay or lesbian*
    - *Completely gay or lesbian*
    - *Other*

Given the very small numbers in some cells, this was recoded into a binary variable (sexual minority) with '*completely heterosexual / straight*' coded as 0 and all other response options coded as 1.

https://doi.org/10.1027/0227-5910/a000951
© 2024 The Author(s). Distributed as a Hogrefe OpenMind article under the license CC BY 4.0 (https://creativecommons.org/licenses/by/4.0)

For 10 participants these data were missing, but they did respond to a question on sexual attraction.

- *I have felt sexually attracted...*
    - *Only to opposite sex*
    - *More often to opposite sex and at least once to same sex*
    - *About equally often to opposite sex and at least once to same sex*
    - *More often to same sex and at least once to opposite sex*
    - *Only ever to same sex*
    - *I have never felt sexually attracted to anyone at all*

For the participants with missing data sexual minority was coded as 0 for the response '*only to opposite sex'* and coded as 1 for all other response options. Data were missing for 0.2% of Wave 7 respondents for whom valid disability and self-harm data were available.

Family Poverty

Family income poverty was assessed at Waves 1-4 of MCS (cohort member ages 9 months, 3 years, 5 years and 7 years). In line with UK government approach to defining income poverty, poverty was defined as having a combined household equivalized income (before housing costs) of less than 60% of the weighted sample median. The modified OECD equivalization scale was used to adjust income considering household composition. Data from all available Waves were combined to create a binary variable: not exposed to income poverty in any Wave vs. exposed to income poverty in one or more Waves). Data were missing for <0.1% of Wave 7 respondents for whom valid disability and self-harm data were available.

Bullied at Ages 7, 11 or 14

Self-report data on exposure to bullying was collected from the children/adolescents at ages 7 (Wave 4), 11 (Wave 5) and 14 (Wave 6) years. From these we created a binary variable (never bullied vs. bullied at one or more Wave). Data were missing for 0.2% of Wave 7 respondents for whom valid disability and self-harm data were available.

Depression at age 14

Short-Form Mood and Feelings Questionnaire, a 13-item questionnaire designed to screen for depression in children and adolescents, was administered at age 14.(1) Two studies have suggested using a cut-off of eight or higher for major depression.(1, 2) Data were missing for 3.1% of Wave 7 respondents for whom valid disability and self-harm data were available.

References

[1] Angold A, Costello EJ, Messer SC, et al. Development of a short questionnaire for use in epidemiological studies of depression in children and adolescents. International Journal of Methods in Psychiatric Research 1995;5:237-249.
[2] Thapar A, McGuffin P. Validity of the shortened Mood and Feelings Questionnaire in a community sample of children and adolescents: A preliminary research note. Psychiatry Research 1998;81:259-268.

https://doi.org/10.1027/0227-5910/a000951
© 2024 The Author(s). Distributed as a Hogrefe OpenMind article under the license CC BY 4.0 (https://creativecommons.org/licenses/by/4.0)

Supplementary Table E1: Adjusted prevalence rate ratios for association of specific functional limitations associated with disability and specific self-harming acts undertaken in the last year

Functional limitation associated with disability	Cut or stabbed self	Burned self	Bruised or pinched self	Taken an overdose of tablets	Pulled out hair	Hurt yourself some other way
Mental health	3.79*** (2.80-5.12)	4.22*** (2.54-7.01)	3.04*** (2.36-3.91)	9.37*** (6.26-14.03)	3.54*** (2.50-5.00)	4.32*** (3.05-6.13)
Learning/understanding	2.86*** (2.25-3.63)	3.73*** (2.47-5.64)	2.73*** (2.24-3.35)	4.73*** (3.26-6.88)	3.52*** (2.62-4.74)	4.44*** (3.07-6.42
Memory	3.00*** (2.21-4.06)	3.17*** (1.86-5.41)	2.67*** (2.05-3.50)	4.60*** (2.62-8.10)	3.32*** (2.20-5.03)	4.22*** (2.66-6.69)
Stamina	2.35*** (1.71-3.22)	2.38** (1.31-4.35)	1.93*** (1.40-2.67)	3.53*** (2.07-6.02)	2.82*** (1.95-4.09)	3.20*** (2.02-5.06)
Social/behavioural	1.91* (1.03-3.51)	2.18* (1.12-4.25)	1.75* (1.09-2.82)	3.48** (1.71-7.08)	1.80 (0.94-3.44)	2.97*** (1.73-5.09)
Dexterity	1.16 (0.58-2.00)	0.84 (0.25-2.81)	1.38 (0.84-2.25)	1.38 (0.47-4.03)	1.89 (0.98-3.63)	1.23 (0.34-4.45)
Hearing	1.29 (0.51-3.27)	2.53 (0.96-6.66)	1.81 (0.98-3.32)	3.00 (0.86-10.53)	1.56 (0.66-3.68)	3.04* (1.24-7.43)
Vision	1.39 (0.59-4.64)	0.54 (0.12-2.47)	1.47 (0.53-4.11)	1.43 (0.34-5.88)	1.75 (0.50-6.14)	1.22 (0.36-4.07)
Mobility	1.27 (0.62-2.59)	0.99 (0.40-2.42)	1.07 (0.58-1.98)	1.31 (0.54-3.16)	1.18 (0.54-2.54)	1.31 (0.57-3.01)

Notes:
* p<0.05, ** p<0.01, *** p<0.001

https://doi.org/10.1027/0227-5910/a000951
© 2024 The Author(s). Distributed as a Hogrefe OpenMind article under the license CC BY 4.0 (https://creativecommons.org/licenses/by/4.0)

Supplementary Table E2: Unadjusted bivariate prevalence rate ratios of potential risk factors for self-harm among adolescents with disabilities

	More limiting disability (reference = less limiting disability)	Female sex (reference = male sex)	Other sexual identify (reference = heterosexual sexual identity)	Minority ethnic status (reference = majority ethnic status)	Family income poverty at age 9 months, 3, 5 or 7 years (reference = no exposure)	Adolescent bullied at ages 7, 11 or 14 years (reference = no exposure)	Adolescent at risk of depression at age 14 (reference = low risk)
Ever hurt themselves on purpose in an attempt to end life:	1.17 (0.79-1.71)	1.99** (1.34-2.95)	1.79** (1.23-2.60)	0.66 (0.40-1.10)	1.71** (1.20-2.44)	1.38 (0.93-2.06)	3.40*** (2.39-4.79)
Participation in specific self-harming acts (including non-suicidal self-harm) In the last year …							
Taken an overdose	1.35 (0.80-2.25)	2.35** (1.37-4.08)	1.71* (1.09-2.69)	0.95 (0.55-1.65)	1.61* (1.01-2.57)	1.97** (1.22-3.20)	3.51*** (2.10-5.89)
Cut or stabbed self	1.14 (0.79-1.64)	2.07*** (1.45-2.96)	2.13*** (1.54-2.94)	0.82 (0.53-1.27)	0.85 (0.64-1.10)	1.25 (0.89-1.73)	2.54*** (1.91-3.39)
Burned self	1.68* (1.01-2.80)	1.65* (1.07-2.57)	2.02** (1.31-3.12)	0.95 (0.51-1.78)	0.96 (0.64-1.45)	1.61* (1.03-2.53)	3.37*** (2.22-5.10)
Bruised or pinched self	1.30 (0.89-1.90)	1.81** (1.29-2.58)	1.99*** (1.43-2.76)	0.61* (0.39-0.93)	0.87 (0.65-1.15)	1.32 (0.94-1.89)	2.06*** (1.61-2.62)
Pulled out hair	1.50 (0.98-2.30)	1.99** (1.28-3.11)	1.66** (1.14-2.49)	0.46* (0.24-0.85)	0.86 (0.61-1.20)	1.32 (0.88-1.97)	2.79*** (1.91-4.09)
Hurt self some other way	1.82* (1.10-2.99)	1.51 (0.94-2.47)	2.05** (1.28-3.28)	1.10 (0.65-1.83)	0.78 (0.50-1.18)	1.53 (0.92-2.48)	2.19*** (1.47-3.24)
Notes: * p<0.05, ** p<0.01, *** p<0.001							

https://doi.org/10.1027/0227-5910/a000951
© 2024 The Author(s). Distributed as a Hogrefe OpenMind article under the license CC BY 4.0 (https://creativecommons.org/licenses/by/4.0)

Supplementary Table E3: Unadjusted bivariate prevalence rate ratios of potential risk factors for self-harm among adolescents without disabilities

	Female sex (reference = male sex)	Other sexual identify (reference = heterosexual sexual identity)	Minority ethnic status (reference = majority ethnic status)	Family income poverty at age 9 months, 3, 5 or 7 years (reference = no exposure)	Adolescent bullied at ages 7, 11 or 14 years (reference = no exposure)	Adolescent at risk of depression at age 14 (reference = low risk)
Ever hurt themselves on purpose in an attempt to end life:	2.42*** (1.74-3.36)	3.54*** (2.59-4.83)	1.02 (0.60-1.73)	1.78*** (1.29-2.44)	2.14*** (1.61-2.84)	4.03*** (2.90-5.61)
Participation in specific self-harming acts (including non-suicidal self-harm) In the last year …						
Taken an overdose	2.08* (1.17-3.70)	2.39** (1.36-4.21)	1.19 (0.55-2.60)	1.34 (0.68-2.64)	1.97* (1.12-3.44)	3.55*** (1.78-7.09)
Cut or stabbed self	1.90*** (1.39-2.60)	3.39*** (2.55-4.51)	0.70* (0.50-0.97)	1.32 (0.99-1.76)	2.19*** (1.66-2.88)	3.68*** (2.99-4.53)
Burned self	1.27 (0.72-2.22)	2.38** (1.43-3.97)	0.60* (0.37-0.99)	1.98* (1.17-3.35)	2.39*** (1.76-3.24)	3.40*** (2.54-4.53)
Bruised or pinched self	1.18 (0.93-1.50)	2.75*** (2.20-3.44)	0.60*** (0.45-0.79)	0.94 (0.72-1.22)	1.69*** (1.33-2.15)	2.71*** (2.26-3.24)
Pulled out hair	1.82** (1.18-2.82)	3.69*** (2.60-5.24)	0.77 (0.49-1.19)	1.26 (0.85-1.85)	2.59*** (1.74-3.84)	4.37*** (3.45-5.54)
Hurt self some other way	0.70 (0.45-1.08)	2.43*** (1.62-3.68)	0.40*** (0.24-0.66)	0.77 (0.46-1.31)	1.38 (0.89-2.15)	3.45*** (2.47-4.81)

Notes: * p<0.05, ** p<0.01, *** p<0.001
